# Comparison of SARS-CoV-2 Detection in Nasopharyngeal Swab and Saliva Samples from Patients Infected with Omicron Variant

**DOI:** 10.3390/ijms24054847

**Published:** 2023-03-02

**Authors:** Licia Bordi, Giuseppe Sberna, Eleonora Lalle, Lavinia Fabeni, Valentina Mazzotta, Simone Lanini, Angela Corpolongo, Anna Rosa Garbuglia, Emanuele Nicastri, Enrico Girardi, Francesco Vaia, Andrea Antinori, Fabrizio Maggi

**Affiliations:** 1 Laboratory of Virology and Biosafety Laboratories, National Institute for Infectious Diseases “L. Spallanzani” IRCCS, 00149 Rome, Italy; 2 Clinical and Research Department, National Institute for Infectious Diseases “L. Spallanzani” IRCCS, 00149 Rome, Italy; 3 Scientific Direction, National Institute for Infectious Diseases “L. Spallanzani” IRCCS, 00149 Rome, Italy; 4 General and Health Management Direction, National Institute for Infectious Diseases “L. Spallanzani” IRCCS, 00149 Rome, Italy

**Keywords:** SARS-CoV-2, saliva samples, COVID-19 diagnosis, monoclonal antibodies, antiviral agents

## Abstract

To compare the detection of the SARS-CoV-2 Omicron variant in nasopharyngeal-swab (NPS) and oral saliva samples. 255 samples were obtained from 85 Omicron-infected patients. SARS-CoV-2 load was measured in the NPS and saliva samples by using Simplexa™ COVID-19 direct and Alinity m SARS-CoV-2 AMP assays. Results obtained with the two diagnostic platforms showed very good inter-assay concordance (91.4 and 82.4% for saliva and NPS samples, respectively) and a significant correlation among cycle threshold (Ct) values. Both platforms revealed a highly significant correlation among Ct obtained in the two matrices. Although the median Ct value was lower in NPS than in saliva samples, the Ct drop was comparable in size for both types of samples after 7 days of antiviral treatment of the Omicron-infected patients. Our result demonstrates that the detection of the SARS-CoV-2 Omicron variant is not influenced by the type of sample used for PCR analysis, and that saliva can be used as an alternative specimen for detection and follow-up of Omicron-infected patients.

## 1. Introduction

COVID-19 has been declared a pandemic by the World Health Organization (WHO), with more than 614 million cases of COVID-19 confirmed [1]. New genetic variants of concern (VOCs) of SARS-CoV-2 emerged, rapidly spreading worldwide, and exhibiting increased transmissibility and/or immune evasion properties that threatened global efforts to control the pandemic. The Alpha (B.1.1.7), Beta (B.1.351), and Gamma (P.1) VOCs disseminated globally and drove epidemic resurgences in many different countries, but the highly transmissible variant that subsequently displaced all the other VOCs in most regions of the world was Delta one [2,3]. In October 2021, however, a new SARS-CoV-2 variant emerged in South Africa, starting to dominate SARS-CoV-2 infections in the world: the Omicron variant (B.1.1.529) [4]. This new variant and descendent lineages drew particular attention due to the high number of mutations (26–32 mutations in the spike protein alone) [5], and the higher transmissibility and immune escape ability, as compared to the Delta variant [6].

Although nasopharyngeal swabs (NPS) have for a long time been considered the gold standard for SARS-CoV-2 detection, scientific evidence has gradually emerged indicating that molecular tests performed on saliva have diagnostic sensitivity and specificity comparable to those observed with NPS [7,8,9,10] and that saliva, due to its easy, painless collection and viral stability represents a suitable alternative sample for SARS-CoV-2 detection [11,12]. Nevertheless, the collection and processing of saliva are undoubtedly poorly standardized procedures, and both can negatively influence SARS-CoV-2 detection [12]. Thus, differently originated saliva samples can be collected, having different characteristics: the posterior oropharyngeal saliva produced when coughing or clearing the throat contains mixed respiratory secretions from upper and lower airways while the oral saliva produced by the salivary glands does not contain respiratory secretions [13]. In one of our previous studies, we described a high concordance in virus detection and quantification between NPS and oral saliva samples, with no significant difference in median Ct values obtained in the two matrices during all follow-up periods [7].

After the Omicron VOC appearance, Marais et al. described altered shedding kinetics in Omicron-infected patients, with a substantial increase in detectable viral RNA in saliva, suggesting that saliva could be the preferred sample type for Omicron variant detection [14], also taking into account the different tropism observed for the B.A.1 and B.A.2 variants as compared to the Delta variant [15]. Considering our experience with the use of saliva as a diagnostic sample during the first phase of the pandemic and throughout the spread of the Delta variant [16,17,18,19], and the few and conflicting studies on this topic [20,21], we decided to investigate the impact of the sample type on the performance of Omicron variant detection, analyzing NPS and saliva samples from patients infected with the Omicron variant and comparing viral loads measured in the two matrices by using different molecular platforms.

## 2. Results

Eighty-five patients were included with a median age of 66 years (range: 23–87 years); 33 of them (38.8%) were females. Patients accessed testing for mild-to-moderate COVID-19 symptoms; the median time from symptoms onset to presentation was 3 days (IQR 2–4). All patients but one were laboratory-confirmed positive for viral RNA at baseline. Genetic characterization of the infecting SARS-CoV-2 variant resulted in 63 (74%) BA.1, 15 (18%) BA.2, and 1 (1%) BA. 4/5 variants. In the remaining patients, no SARS-CoV-2 variant was determined, likely due to low infecting viral load.

We first compared the performance of the Alinity mSARS-CoV-2 AMP and the Simplexa™ COVID-19 direct assays in detecting SARS-CoV-2 RNA in 255 NPS and saliva samples coming from these 85 patients.

A total of 142/255 saliva samples (55.7%) reacted positive for SARS-CoV-2 RNA, while 113/255 were negative (44.3%), with Simplexa reference method. As far as NPS is concerned, a total of 157/255 (61.5%) resulted SARS-CoV-2-positive and 98/255 (38.4%) negative. When comparing these results with those obtained with Alinity assay, 135 saliva samples resulted positive with both methods and 98 negative, showing 91.4% inter-assay concordance, with an almost perfect agreement (κ = 0.824; 95% CI = 0.754–0.894). Considering NPS, 153 samples were positive with both assays while 57 were negative, showing 82.4% inter-assay concordance, with moderate agreement (κ = 0.599; 95% CI = 0.499–0.699).

Moreover, a significant correlation between Ct values measured by the two methods was observed both in saliva (r = 0.9545, *p* < 0.0001) and in NPS samples (r = 0.9459, *p* < 0.0001).

**Figure 1:**
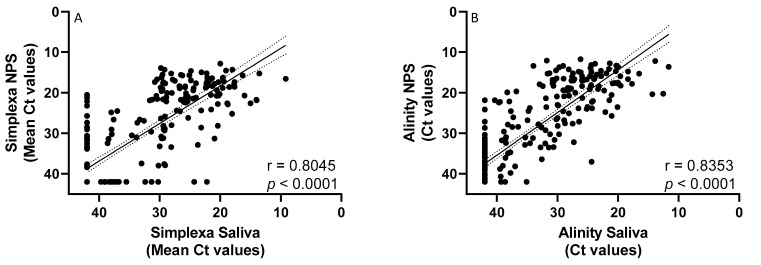
Correlation between Ct values obtained in NPS vs. Ct values obtained in saliva samples using Simplexa and Alinity platform. (**A**) Correlation between Ct values obtained in NPS vs. Ct values obtained in saliva samples using Simplexa; (**B**) Correlation between Ct values obtained in NPS vs. Ct values obtained in saliva samples using Alinity platform. Linear regression with 95% of Confidence Interval (dashed line). For statistical calculations, an arbitrary value of 42 Ct was assigned to negative samples for both assays; results obtained by Simplexa are expressed as the mean of Ct obtained from single genes ORF1ab and S.

Secondly, linear regression analysis showed a statistically significant correlation between Ct values measured in NPS versus saliva samples either using Simplexa (r = 0.8045, *p* < 0.0001) or Alinity platform (r = 0.8353, *p* < 0.0001) (Figure 1A,B).

**Figure 2:**
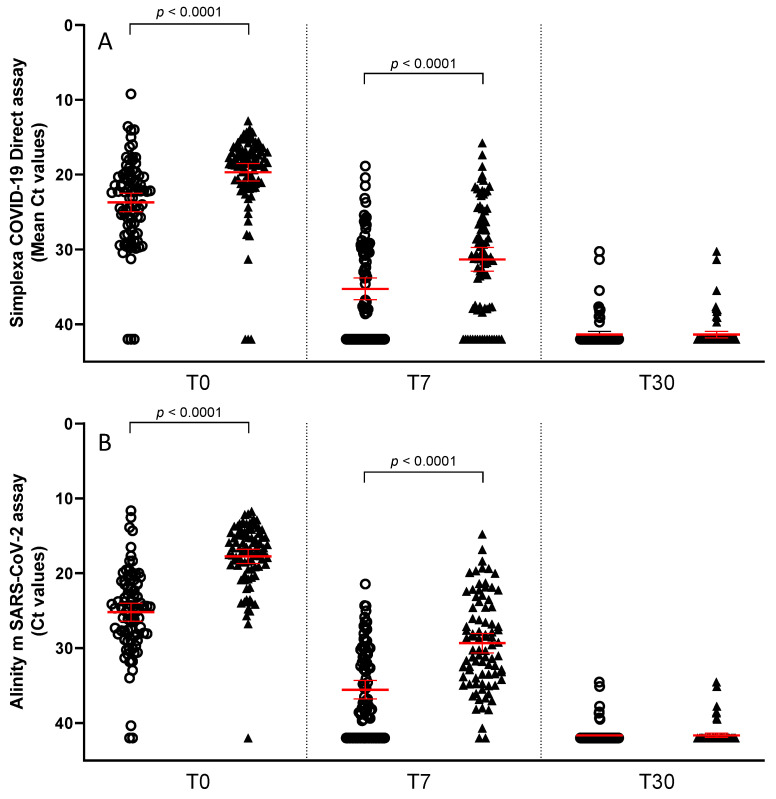
Cycle threshold values of SARS-CoV-2 RNA in 85 saliva and NPS samples at T0, T7 and T30 after pharmacological treatment. Ct values obtained in saliva samples are represented with circles, while those obtained in NPS are represented with triangles. Median Ct values with 95% of Confidence Interval are indicated with red lines. Statistically significant differences were found in Ct values of the two matrices either T0 or T7 by paired T-test both using Simplexa (**A**) and Alinity (**B**) platforms.

**Figure 3:**
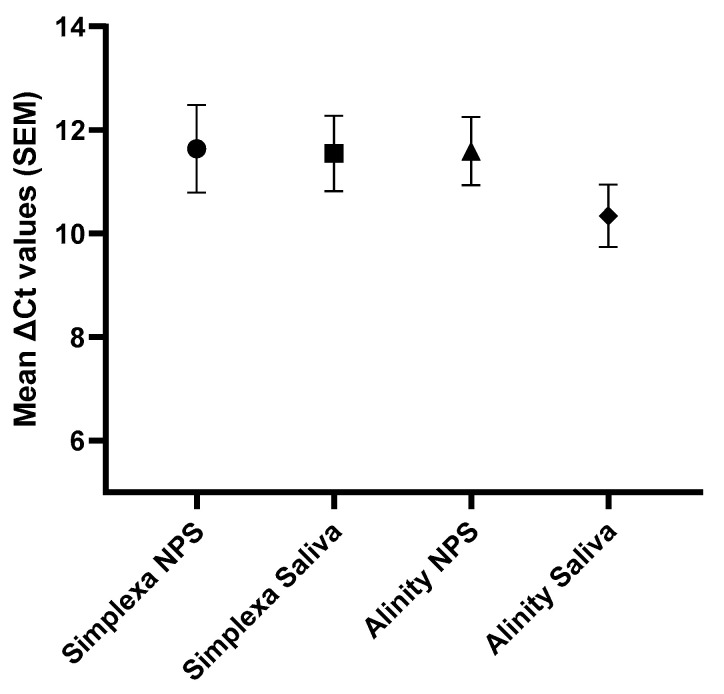
Mean Cycle threshold values drop after 7 days of treatment in saliva and NPS samples. Results are expressed as mean ΔCt (Ct T7–Ct T0), obtained using both Simplexa and Alinity methods.

Finally, the SARS-CoV-2 loads measured in NPS and saliva samples at baseline (T0) and days 7 (T7) and 30 (T30) post-SARS-CoV-2 antiviral agents or monoclonal neutralizing antibodies treatment were compared. Median Ct values were significantly lower in NPS than in saliva samples both at T0 (19.7 vs. 23.7, *p* < 0.001) and T7 (31.3 vs. 35.3, *p* < 0.001) using the Simplexa assay. Comparable results were obtained using the Alinity platform (T0: 17.8 vs. 25.2 Ct, *p* < 0.001; T7: 29.3 vs. 35.5 Ct, *p* < 0.001). No difference among the matrices was seen at T30, probably due to the negative SARS-CoV-2 detection in most tested samples (Figure 2A,B). Again, no difference was observed when the mean Ct drop at day 7 (ΔCt: Ct T7–Ct T0) was calculated (Figure 3). In detail, ΔCt was 11.6 and 11.5 in NPS and saliva samples, respectively, by Simplexa assay, and 11.6 and 10.3 by Alinity, thus demonstrating that the drop in SARS-CoV-2 level was similar using the two biological matrices.

## 3. Discussion

Saliva has more recently entered the shortlist of clinical samples to which the current laboratory SARS-CoV-2 tests can be applied. However, although there is increasing evidence of comparable sensitivity and specificity with respect to NPS, concerns still exist about the diagnostic use of saliva for SARS-CoV-2 detection [8,9,10], and caution must be still exercised when using it.

In the present study, 255 NPS and oral saliva samples from 85 patients infected with the Omicron variant were investigated using two molecular platforms for SARS-CoV-2 presence and quantification. The platforms were chosen because they were different in their methodologic approaches: the Simplexa platform allows for fast results directly from NPS and saliva specimens, eliminating the traditional RNA extraction step and amplifying the ORF1ab and S genes of the viral genome. The Alinity platform performs automatic sample extraction, amplification of two different targets (SARS-CoV-2 RdRp and N genes), detection, and result calculation in under two hours.

Interesting findings result from our study. First, very good inter-assay concordance between the two diagnostic platforms was revealed, both for oral saliva (91.4%) and NPS (82.4%) samples, with results suggesting the best performances of Simplexa and Alinity systems in detecting SARS-CoV-2 RNA in saliva and NPS, respectively. Nevertheless, a highly significant correlation was observed among Ct values measured by Simplexa versus those revealed by Alinity both in saliva and in NPS samples. Again, Ct values obtained in NPS and saliva samples using both platforms significantly correlated thus indicating a good concordance between the two matrices, in agreement with other studies [20,22,23].

Second, when the amount of SARS-CoV-2 RNA in saliva was compared with that in NPS at the different time points of collection, the median Ct value in NPS was statistically lower than that in saliva both at T0 and T7. These data are in line with results obtained by Migueres et al. showing that, independently from the presence/absence of symptoms, NPS samples from Omicron positive patients have lower Ct values with respect to saliva [24]. Similarly, in the study of Cornette et al., although saliva is proposed as specimen with higher detection rate, lower NPS Ct values are reported [21]. In contrast, Marais et al. described that the Omicron variant might be more readily detected using RT-PCR in saliva swab due to an increase of viral RNA as compared to paired mid-turbinate swabs [14]. In this respect is important to consider that our results are referred to oral saliva, spontaneously produced without external stimuli and collected by passive drooling, not after coughing. This different type of collection could contribute to explaining the conflicting results obtained, together with the fact that in our study NPS were analyzed, and not mid-turbinate swabs.

Third, looking at the mean Ct reduction after 7 days of treatment with antiviral agents or monoclonal neutralizing antibodies, no difference was observed in NPS versus saliva samples, thus suggesting a comparable viral load reduction after treatment with antiviral or monoclonal antibodies between the two matrices.

Overall, our results confirmed high concordance among Ct values obtained in saliva and NPS samples by two molecular platforms, although lower viral loads in saliva than in NPS were observed. Nevertheless, the mean of post-treatment Ct drop was similar in saliva and NPS samples, thus indicating that these samples can be equivalently used for the detection of the Omicron variant, also during follow-up studies. Even if some specimens become virus-negative during the last stages of treatment, the viral decline could still be accurately assessed over time in saliva samples. A limitation of this study is the non-homogeneous number of variants analyzed, thus not allowing to evaluate if the post-treatment Ct drop is equally maintained in saliva and NPS samples in all Omicron variants. Further investigation could elucidate this point and Next Generation Sequencing could be performed to distinguish variant BA.4 from BA.5 and to allow the characterization of different Omicron sublineages.

## 4. Materials and Methods

### 4.1. Study Population and Sample Collection

**Figure 4:**
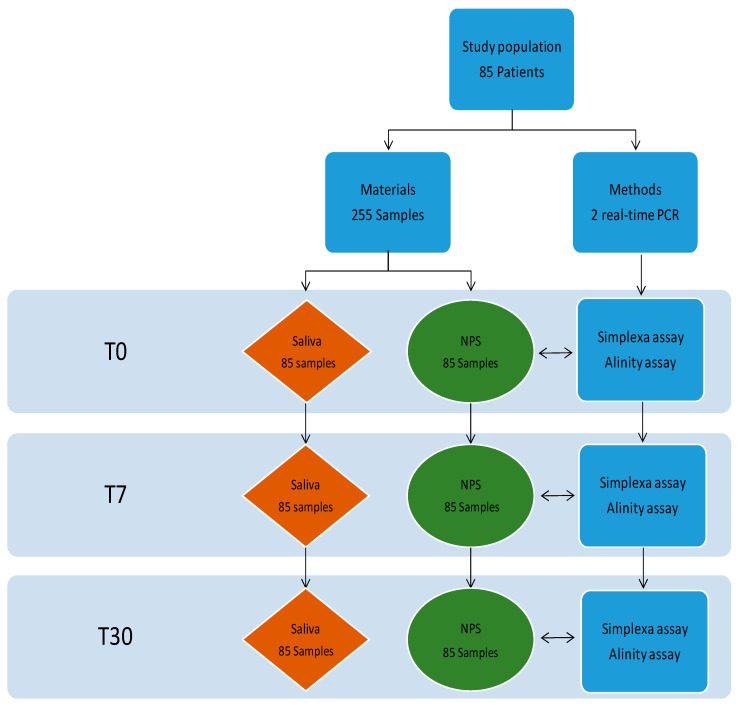
Schematic workflow of the study.

Simultaneous NPS and saliva samples were collected from outpatients who accessed the National Institute for Infectious Diseases “L. Spallanzani” from March to May 2022, for treating mild-to-moderate COVID-19, which had been onset for less than 5 days. No patient was hospitalized. The positivity was confirmed in our laboratory for all but one samples. All patients were longitudinally tested at three-time points: at baseline immediately before using direct antiviral agents or monoclonal neutralizing antibodies treatment (T0), and at day 7 (T7) and day 30 (T30) post-treatment initiation, as schematically summarized in Figure 4. As far as the treatment is concerned, 28 (32.9%) patients received Tixagevimab/cilgavimab, 24 (28.2%) Nirmatrelvir/ritonavir, and 33 (38.8%) Sotrovimab.

NPS was put into a sterile tube containing 2–3 mL of viral transport media (Copan UTM^®^ Universal Transport Medium, Copan Diagnostics Italia s.p.a., Brescia, Italy), and at least 2 mL of saliva were self-collected, under medical personal supervision, by passive drooling, spontaneously produced without external stimuli, in a sterile container without any buffer added, at least 30 min after drinking or eating or washing teeth. All samples were processed and analyzed immediately after collection without being frozen. Data from biological samples were used after complete anonymization only.

### 4.2. SARS-CoV-2 Real-Time PCR and Sequence Analysis

NPS and saliva samples were processed twice, each sample being tested using two different molecular platforms: the Alinity mSARS-CoV-2 AMP assay (Abbott Diagnostics GmbH, Wiesbaden, Germany), targeting RdRp and N genes, and the Simplexa™ COVID-19 direct assay (DiaSorin Molecular LLC, Cypress, CA, USA) targeting S and ORF1ab genes.

For the Simplexa™ COVID-19 Direct assay, one vial of reaction mix was thawed for each sample followed by loading 50 μL of NPS and 50 μL of reaction mix on a direct amplification disk onto the LIAISON^®^ MDX instrument. As far as the saliva sample is concerned, it was previously diluted 1:1 with 0.9% NaCl.

For the Alinity mSARS-CoV-2 AMP assay, NPS samples were directly loaded on the Alinity instrument using Lysis solution, while saliva samples were previously diluted with Alinity m Specimen Diluent with saliva: diluent volume ratio of 1:1.25.

To genetically characterize the SARS-CoV-2 variants, saliva and/or NPS samples tested virus-positive by real-time PCRs were sequenced by the Sanger method. Viral RNA was extracted by the automated extraction system QiaSymphony (Qiagen Instruments AG Switzerland, Hilden, Germany), according to the manufacturer’s instructions. A fragment corresponding to the aminoacidic coverage 399–616 of the SARS-CoV-2 Spike gene was amplified by one-step RT-PCR kit (Qiagen).

The amplified products were sequenced by the Big Dye Terminator Cycle Sequencing kit v3.1 (Applied Biosystems, Foster City, CA, USA) and an automatic DNA sequencer (ABI model 3130 and/or 3500 XL, Applied Biosystems, Foster City, CA, USA). The sequences were compared by alignment with the original Wuhan virus sequence (accession number: NC_045512.2), as previously described [25].

The fragment used to define variants was sufficient to discriminate variants BA.1 from BA.2 and from BA.4/5. In fact, considering the sequenced portion of the S gene, variant BA.2 differs from BA.1 in the absence of substitutions G446S, G496S AND T547K, and in the addition of two substitutions D405N and R408S. Variants BA.4 and BA.5 were different from the other two, mainly from the presence of mutation L452R and F486V.

### 4.3. Statistical Analysis

Data management and analyses were performed using GraphPad Prism version 9.3.1 (GraphPad Software, La Jolla, CA, USA). Descriptive analysis was performed to characterize patients enrolled in the study and above described. The evaluation of the concordance of the qualitative results was assessed considering Simplexa™ COVID-19 Direct assay as a reference test based on previous reports [7,16], and using the weighted Cohen Kappa statistic and its 95% confidence interval (CI). Linear regression analysis and Student t-test were performed to evaluate the relationship between quantitative results and the difference among results, respectively. A *p* ≤ 0.05 was considered statistically significant in all tests. To perform statistical analyses, an arbitrary value of 42 cycle threshold (Ct) was assigned to negative samples for both assays. Again, results obtained by Simplexa were expressed as the mean of Cts obtained from ORF1ab and S genes, thus making possible the result compared with the Alinity method. This latter method does not allow for discriminating Ct values for each gene (RdRP and N genes) but it provides a single, mathematically calculated result.

## Data Availability

The data presented in this study are available on request from the corresponding author. The data are not publicly available due to privacy restrictions.
